# The Mediating Role of Dysfunctional Coping in the Relationship between Beliefs about the Disease and the Level of Depression in Patients with Rheumatoid Arthritis

**DOI:** 10.1155/2014/585063

**Published:** 2014-01-19

**Authors:** Michal Ziarko, Ewa Mojs, Bartosz Piasecki, Wlodzimierz Samborski

**Affiliations:** ^1^Adam Mickiewicz University, Institute of Psychology, 89/AB Szamarzewskiego Street, 60-568 Poznan, Poland; ^2^Department of Clinical Psychology, Poznan University of Medical Sciences, 70 Bukowska Street, 60-812 Poznan, Poland; ^3^Department of Physiotherapy, Rheumatology and Rehabilitation, Poznan University of Medical Sciences, 135/147 28 Czerwca 1956r. Street, 61-545 Poznan, Poland

## Abstract

*Aim.* Rheumatoid arthritis is one of the most severe chronic diseases. In many cases it leads to disability and results in a decreased quality of life and increased levels of anxiety and depression. The problem that needs to be addressed is the following: which mental processes lead to increased levels of depression in patients with rheumatoid arthritis? *Methods.* 210 patients with rheumatoid arthritis hospitalized in rheumatology wards took part in the research. They filled in illness perception questionnaires (IPQ-R) and questionnaires for testing strategies of handling stress (Mini-COPE) and the level of depression (CES-D). *Results.* The observed correlation coefficients indicate that several elements of the perception of one's disease moderately contribute to a high level of depression. Moreover, frequent use of dysfunctional coping strategies contributed to high levels of depression. Dysfunctional coping was moderately linked to depression. *Conclusion.* The conducted analyses confirmed the links between the beliefs about the disease and levels of depression and showed that the use of dysfunctional coping strategies mediates the relationship between the following elements of the representation of the disease: *illness coherence, emotional representation, psychological attribution, risk factors, *and *the level of depression*.

## 1. Introduction

Chronic diseases are one of the most serious health problems faced by the population of developed countries. They are diseases of long duration, often incurable, through which people mostly struggle with all their lives [1]. Being chronically ill always leads to a number of consequences which impact most of the aspects of one's life, whereas an acute disease does not usually negatively impact the quality of life of the sick [2]. 

Rheumatoid arthritis (RA) is a chronic, progressive, autoimmune disease characterised by an inflammation of the synovium in joints. Inflammation leads to the damage of articular and periarticular tissues and the deformation and joint dysfunction. In Caucasians, RA is diagnosed in 1 in 100 people, and it affects women 3 to 4 times more often than men. While the disease progresses, patients face numerous somatic, psychological, and social dysfunctions. The coexistence of other diseases, including other chronic diseases, complications related to treatment, chronic pain and fatigue, and deficits in mobility, leads to patients eventually losing their ability to function independently. 1/3 of patients became disabled as a result of the progressing pathological processes [3].

The occurrence of negative effective states is an important aspect of the functioning of patients with RA strongly impacting on their quality of life. RA patients experience anxiety and depressive symptoms more often than the general population. It is estimated that depression occurs in 14–62% of ill patients [4–7]. A high percentage of depression among RA patients can be explained with two hypotheses. According to the first one, the impact of continuing confrontation with one's own powerlessness and helplessness facilitates the occurrence and maintenance of depressive symptoms in patients with RA. Another hypothesis sees sources of depression in neuroimmunobiological mechanisms, with the dominant role of proinflammatory cytokines, which consequently lead to a disruption in the functioning of the serotonergic system [8].

The relationship between depression and RA can often become a vicious circle. Depression worsens the somatic symptoms of RA, decreases patient's motivation to take an active part in treatment, and, as a result, negatively influences the efficiency of therapeutic interventions. Increased activity of RA in turn affects the intensification of depressive symptoms and makes therapy more difficult. People suffering from depression are exposed to a higher risk of recurring severe pain, development of the disease, greater degree of joint deformation, and reduced physical, social, and professional activity. They are also characterised by a greater dependency on healthcare, which is manifested in more frequent medical appointments and hospitalisations [8, 9].

It is argued that from the moment of diagnosis, sick people create their own cognitive representation of a disease. This phenomenon is known as the image of one's disease, the attitude towards one's disease, or the beliefs about one's disease [10, 11]. According to Leventhal et al. the beliefs of one's disease include six aspects [12]. They are (a) the nature of the disease (*identity*); (b) the beliefs regarding the cause of the disease; (c) predictions about the course of a disease (*timeline cyclical*) and its duration (*timeline acute/chronic*); (d) predictions related to the consequences of a disease (*consequences*); (e) beliefs about the possibility of controlling the symptoms (*personal control*) and the treatment process (*treatment control*); (f) a sense of coherence (*illness coherence*). These aspects of cognitive representation of a disease have been enriched by a seventh dimension connected with emotional responses (*emotional representations*) to a disease, which regards emotions evoked while becoming ill [13]. 

The representation of one's illness equates the assessment of a stressful situation [14]. Leventhal attributes the function of regulating the behaviour to the representation of a disease [11, 12]. This means that depending on the beliefs held by patients about their disease, he/she will behave differently, which might influence their health. According to this assumption, a perception of a disease codecides the diversity and variation of the response to becoming ill. 

This assumption has been confirmed by studies of patients suffering from various chronic diseases. For example, there is a relationship between an image of one's illness and a sense of quality of life in patients suffering from diabetes [15], as well as the seriousness of symptoms of rheumatoid arthritis a year after initial diagnosis [15–17]. Among a group of patients suffering from ischemic heart disease, the beliefs of patients regarding the disease predict subsequent health behaviours, involvement in cardiac rehabilitation, and return to performing social, and especially, professional roles [18–20]. In a meta-analysis of Leventhal's model, Hagger and Orbell indicate that the lack of focus on the consequences of a disease and not concentrating on its symptoms lead to better adaptation to living with a chronic disease which can be understood as fulfilment of social roles, small physiological consequences of the disease, the level of well-being, and vitality [21]. The perception of the causes, however, had no relevance to the level of patients' adjustment to their health situation.

When it comes to RA it has been observed that the beliefs held about one's disease can predict the severity of symptoms a year after the initial diagnosis, as well as the sense of exhaustion with the disease [16, 17, 22]. It has also been shown that, in patients who have negative beliefs about their illness, the course of RA is more serious and the disease is characterised by more activity. This situation has been mainly linked to experiencing stronger pain, a higher level of disability, and a worse mental condition [23]. A link has also been made between the representation of the disease and the psychological well-being of patients with RA [24].

In relational theories of stress it is assumed that cognitive assessment (in the case of a chronic disease assessment involves creating representations of an illness) triggers an activity described as *coping*, which is generally understood as “a response to a stressful or negative incident” [25]. Ways of classifying coping strategies have been identified. They include coping focused on the problem versus coping focused on the emotions or approach versus avoidance coping [26, 27]. One of the most extensive classifications of coping is proposal of Scheier and Carver, who distinguish fourteen coping strategies, including active coping, planning, seeking instrumental social support, seeking emotional social support, avoidance, return to religion, positive reevaluation, refraining from action, acceptance, concentration on emotions, denial, diverting attention, cessation of operations, use of alcohol and/or drugs, and a sense of humour [28]. Wong et al. suggest the possibility of assigning the coping strategies distinguished by Scheier and Carver into three groups: coping focused on the problem, coping focused on emotions, and dysfunctional coping [29].

Much research dedicated to coping with stress deals with determining which strategies encourage positive adaptation to the disease and which of them impede it. With regard to rheumatoid arthritis patients it has been found that the use of the strategies referred to as active or focusing on the problem leads to a better functioning manifested as a positive effect, whereas the use of passive strategies or focusing on emotions results in a higher probability of depression, pain, and functional maladjustment [30, 31]. The research on coping with a chronic disease ascribes a mediating function between the assessment of the disease and the effects of coping with a disease or between an assessment of a disease and its consequences [32].

Because one of the major problems RA patients deal with is increased levels of depression, there is an interesting problem to address, Which factors lead to high levels of depression and what kind of interaction is there between them? In order to solve this problem a research model was proposed where beliefs about the disease were treated as an independent variable and the intensity of depression as a dependent variable.

Dysfunctional coping was considered a potential factor mediating the relationship between beliefs about the disease and the level of depression.

## 2. Data and Methodology

### 2.1. Subjects

210 people with rheumatoid arthritis took part in the research. Respondents were between 25 and 86 years old (M = 54.92; SD = 14.85). Generally 12.4 years had passed since the diagnosis of the disease (SD = 10.12; MIN = 0.1; MAX = 47). The examined group consisted of mainly women, which is in line with epidemiological data. There were 176 females (76.7%). There were no differences between women and men in terms of age (M_*M*_ = 55.14; SD_*M*_ = 13.37; M_*K*_ = 54.85; SD_*K*_ = 15.31; *t* = −0.129; *P* = 0.897) and the duration of the disease (M_*M*_ = 13.16; SD_*M*_ = 10.49; M_*K*_ = 12.16; SD_*K*_ = 10.02; *t* = 0.583; *P* = 0.561). Participants in the study were hospitalised in rheumatology wards. The research was conducted during a period of patients' health deterioration, while they were in need of being hospitalised because of this fact. Participation in the study was voluntary and anonymous.

### 2.2. Tools

The participants individually completed questionnaires measuring the intensity of depression, beliefs about the disease, and coping strategies [13, 33, 34]. The CES-D scale *(Center for Epidemiologic Studies on Depression)* was used for estimating the level of depression a week before the study [33]. It consisted of 20 statements related to depressed mood, feelings of guilt, worthlessness, and somatic symptoms. The task of the participants was to assess how often they felt these symptoms. Answers were given according to a four-point scale, for which the marginal points were used as follows: 0 was used for “rarely” or “never” (less than 1 day) and 3 was for “most of the time” or “all the time” (5–7 days).

Mini-COPE, Stress Coping Inventory [34, 35], is a questionnaire that consisted of 28 statements allowing for measuring 14 strategies of coping with a difficult situation. They included active coping, planning, positive reevaluation, acceptance, a sense of humour, the return to religion, seeking emotional support, seeking instrumental support, substitute activities, denial, discharge, substance use, cessation of operations, and blaming oneself. Answers were given according to a four-point scale, for which the marginal points were used as follows: 0-I never do it and 4-I always do it. The results were calculated as the sum of responses on a particular subscale. Due to the large amount of measured coping strategies, in order to carry out general analyses, the number of coping strategies should be reduced. Coolidge et al. indicate that the distinguished coping strategies can be classified into three groups: coping focused on the problem (active coping, planning, and seeking instrumental support), coping focused on emotions (positive reevaluation, acceptance, a sense of humour, the return to religion, and seeking emotional support), and dysfunctional coping (activities, denial, discharge, substance use, cessation of operations, and blaming oneself) [36]. In the presented research the results obtained by patients on a scale of dysfunctional coping were taken into account. The Revised Illness Perception Questionnaire IPQ-R is used for studying cognitive representations of a disease [13, 37]. It consists of three parts. The first one is used to measure the perceived symptoms, the second one measures beliefs held by a patient about various aspects of the disease, and the third section refers to the causes of the disease as perceived by the patient. In the presented research the data obtained in the second section were used. They particularly refer to the perception of the disease as (1) being acute or chronic, (2) being characterized by recurrent relapses, (3) having a variety of consequences, (4) being under personal control, and (5) causing a variety of emotional states. Moreover, this section allowed for measuring beliefs concerning (6) the ability to control one's health due to treatment and (7) the level of understanding the disease. The study also used data from the third section, which comprised the perceived psychological causes of the disease and active risk factors leading to it. Satisfactory reliability coefficients were obtained only for both of those subscales.

Answers were given according to a four-point scale, for which the marginal points were used as follows: 1-I definitely disagree and 5-I definitely agree.

### 2.3. Statistical Procedures

When preparing the data for analysis, the gaps in the data were filled with the use of the EM procedure. The responses were excluded from data analysis when they lacked more than 2% of the answers. Firstly, descriptive statistics of the measured variables, as well as the *α*-Cronbach reliability coefficients of individual questionnaires, were calculated (see Table 1). In order to verify the relationship between various elements of the representation of the disease and the intensity of depression and dysfunctional coping, *r*-Pearson correlation coefficients were counted. In order to test the hypothesis about the mediating role of dysfunctional coping in a relationship between a representation of a disease and the intensity of depression, an analysis of a simple moderation as recommended by Preacher and Hayes was carried out [38, 39].

## 3. Results

### 3.1. Descriptive Statistics and Correlation Coefficients between the Representation of the Disease and the Intensity of Depression

Table 1 shows descriptive statistics of the measured variables and the correlation coefficients between them. Given the stated problem, the perception of relationships between the perception of the disease and dysfunctional coping with the level of depression are very interesting. The observed correlation coefficients indicate that the following elements of the perception of one's disease moderately contribute to the high level of depression: the perception of their illness in terms of being acute rather than chronic (*r* = −0.14*), not seeing the possibility of influencing the process of treatment (*r* = −0.20**), lack of understanding of one's illness (*r* = −0.30**), experiencing many negative emotions such as anger, anxiety, and feelings of helplessness (*r* = 0.42**), and indicating that psychological factors are responsible for the development of the disease (*r* = 0.25**) and risk factors (*r* = 0.17*). Moreover, frequent use of dysfunctional coping strategies contributed to high levels of depression (*r* = 0.33**). Dysfunctional coping was moderately linked to depression (*r* = 0.40**).

### 3.2. The Mediating Role of Dysfunctional Coping Strategies in the Relationship between the Representation of the Disease and the Level of Depression

**Figure 1:**
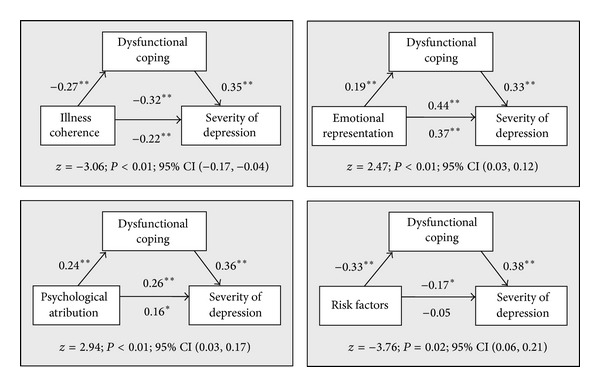
The mediating impact of dysfunctional coping in the relationship between belief about the disease and severity of depression. *P < 0.01 and **P < 0.05.

The conducted analysis showed that dysfunctional coping mediates the relationship between four dimensions of the representation of the disease and the level of depression (see Figure 1). The mediating role of dysfunctional coping was observed in the case of the relations linking *illness coherence, emotional representation, psychological attribution, and risk factors *with the intensity of depression. The mechanism linking *illness coherence *and *risk factors *with depression through dysfunctional coping strategies is similar. People with a high level of *illness coherence *and *risk factors *rarely use dysfunctional coping strategies, which results in a lower level of depression. People who are characterised by a strong emotional reaction to the disease and who perceive the causes of the disease as resulting from psychological factors often tend to use dysfunctional coping strategies, which leads to an increased level of depression in these patients.

## 4. Discussion

The conducted analysis confirmed the significance of some elements of one's self-image and dysfunctional coping to the level of depression in people with rheumatoid arthritis. The protective function, in relation to the level of depression, was identified as the perception of one's disease as a transitional state, the perception of opportunities of active involvement in the process of treatment, understanding it, not having a very strong emotional response to becoming ill (mostly not experiencing emotions such as anger, fear, and helplessness), and not treating one's illness as a consequence of risk factors or psychological factors. This corresponds with the results of a two-year longitudinal study conducted on a group of 52 women with rheumatoid arthritis [40]. It has been observed that a high level of identification with the disease and perception of the consequences of the disease as more serious were linked to a worse physical functioning, stronger pain, and higher levels of anxiety and depressive symptoms. A lower level of control over the disease and a lower belief in the possibility of treatment is connected to a stronger pain and intensified depression. This dependency confirms the assumption that the beliefs about a disease are one of the determinants of health of chronically ill patients.

Moreover, it has been observed that dysfunctional coping increases the risk of depression. It affects the level of this variable directly and indirectly. Its indirect effect is revealed when it is treated as a mediator in the relationship linking various elements of beliefs about the disease with the level of depression. The results obtained for the presented study allow for a better understanding of the mechanism of dysfunctional coping. Dysfunctional coping is regarded as a factor impeding adaption to a chronic disease [30, 31]. The results of the study showed that patients with high levels of dysfunctional coping were characterized by a higher level of depression. This relationship was weakened under certain conditions and intensified under others. If patients feel that they understand their disease and perceive its causes as resulting from risk factors, the likelihood of using dysfunctional coping strategies decreases, which contributes to a lower level of depression. If patients respond to the disease with strong emotions and see its determinants among psychological factors, the likelihood of using dysfunctional coping strategies increases, which contributes to a higher level of depression.

The results obtained have practical implications as they outline potential areas of psychological therapy aimed at improving the functioning of rheumatoid arthritis patients. In such patients, in addition to activities aimed at developing coping skills and living with pain, which is the primary purpose of psychological work, interventions that intend to deal with depression are desirable [41]. It has been observed that experiencing an episode of depression increases the intensity of pain in the future [42]. In the light of the obtained results, this effect can be achieved by including working on one's perception of the disease as part of a programme of treatment. Such types of actions may contribute to a less frequent resorting to dysfunctional coping strategies, which may in turn result in a reduced level of depression among these patients. 

## Figures and Tables

**Table 1:** Descriptive statistics, *α*-Cronbach reliability coefficients, and values of correlation coefficients between the measured variables.

	M	SD	*α*	(1) (a)	(1) (b)	(1) (c)	(1) (d)	(1) (e)	(1) (f)	(1) (g)	(1) (h)	(1) (i)	(2)
(1) Beliefs about the disease													
(a) Acuteness-chronicity	23.05	4.22	0.84										
(b) Consequences of the disease	22.59	4.01	0.73	0.35**									
(c) Self-control	17.94	3.60	0.71	−0.19**	−0.13								
(d) Treatment control	16.44	2.99	0.65	−0.16*	−0.17*	0.30**							
(e) Sense of coherence	15.57	3.66	0.73	0.15*	−0.11	−0.09	0.07						
(f) Cyclicality of symptoms	14.14	2.77	0.74	0.13	0.30**	0.02	0.12	−0.27**					
(g) Emotional representation	20.91	5.15	0.86	0.09	0.32**	−0.02	−0.11	−0.35**	0.17*				
(h) Psychological causes	15.64	4.80	0.79	−0.23**	0.01	0.16*	−0.02	−0.28**	0.19**	0.25**			
(i) Risk factors	16.78	4.83	0.75	−0.25**	−0.01	0.06	0.01	−0.35**	0.14*	−0.04	0.55**		
(2) Level of depression	22.58	12.05	0.92	−0.14*	0.07	−0.03	−0.20**	−0.30**	0.08	0.42**	0.25**	−0.17*	
(3) Dysfunctional coping	13.38	5.37	0.84	−0.13	0.09	0.08	−0.11	−0.25**	0.13*	0.19**	0.23**	0.33**	0.40**

*P < 0.05 and **P < 0.01.
